# Revisiting caspases in sepsis

**DOI:** 10.1038/cddis.2014.488

**Published:** 2014-11-20

**Authors:** M Aziz, A Jacob, P Wang

**Affiliations:** 1 Center for Translational Research, The Feinstein Institute for Medical Research, Manhasset, NY, USA; 2 Department of Surgery, Hofstra North Shore-LIJ School of Medicine, Manhasset, NY, USA

## Abstract

Sepsis is a life-threatening illness that occurs due to an abnormal host immune network which extends through the initial widespread and overwhelming inflammation, and culminates at the late stage of immunosupression. Recently, interest has been shifted toward therapies aimed at reversing the accompanying periods of immune suppression. Studies in experimental animals and critically ill patients have demonstrated that increased apoptosis of lymphoid organs and some parenchymal tissues contributes to this immune suppression, anergy and organ dysfunction. Immediate to the discoveries of the intracellular proteases, caspases for the induction of apoptosis and inflammation, and their striking roles in sepsis have been focused elaborately in a number of original and review articles. Here we revisited the different aspects of caspases in terms of apoptosis, pyroptosis, necroptosis and inflammation and focused their links in sepsis by reviewing several recent findings. In addition, we have documented striking perspectives which not only rewrite the pathophysiology, but also modernize our understanding for developing novel therapeutics against sepsis.

## Facts

- Sepsis refers to an abnormal host immune response against invading pathogens. Despite intense efforts, sepsis still remains a critical problem with significant morbidity and mortality, reflecting the annual hospital care cost of about $16.7 billion.
- Apoptosis and inflammation are the two most highly focused current areas of active investigation concerning pathogenesis in sepsis. Caspases have major roles in apoptosis, inflammation, pyroptosis and necroptosis. It is necessary to update our understanding by revisiting the latest innovations on caspases in sepsis.
- Utilizing caspase inhibitors provide beneficial outcome against sepsis.

## Open Questions

- What are the latest innovations and controversies surrounding the role of caspases in sepsis?
- Are all caspase-deficient mice entirely protective against sepsis?
- What are the potential pitfalls while targeting caspases for the treatment of sepsis?

## Introduction

The critical involvement of a cysteine protease, cell-death abnormality-3 gene family member, in programmed cell death or apoptosis was first discovered in the nematode.^1^ Since then compelling evidence shows that in higher organisms apoptosis is executed by a family of cysteine proteases, known as caspases, that cleave after an aspartate residue in their substrates.^2, 3^ At the same time it was found that other members of the caspase-family, in particular interleukin-1*β*-processing enzyme (interleukin-1*β* converting enzyme, also known as caspase-1), are important for pro-inflammatory cytokine processing.^4, 5^ In addition to apoptosis and cytokine release, recent reports implicate caspases in various cellular events that are summarized in Table 1. Importantly, caspases have been recognized as important factors in human diseases where excessive apoptosis and uncontrolled inflammation are hallmarks of pathology.

Sepsis refers to severe systemic inflammation in response to invading pathogens.^6^ Based on the latest epidemiological survey, about 750 000 cases of severe sepsis per year occur in the USA alone, with an annual mortality estimated at 210 000.^7^ During the initial phase of sepsis, a vigorous induction of the innate immune system can cause exaggerated production of pro-inflammatory cytokines, chemokines and other inflammatory mediators.^6^ Conversely, patients may proceed to an immunosuppressive state, which is characterized by the profound loss of immune reactive cells as well as the induction of tolerance. This process has been termed compensatory anti-inflammatory response syndrome.^6, 8^

Since initial hyperinflammation and late immunosuppression due to excessive cellular apoptosis feature in sepsis, it is worthwhile to rethink the relevance of caspases in both of these events. Also, caspase inhibitors as well as caspase deficiency greatly improve the survival and overall disease outcome in sepsis models.^9^ In this review we summarize key findings of caspases in sepsis, which not only define their role in pathophysiology but also help implement potential therapeutic strategies against this deadly clinical syndrome. We therefore aim to revisit and update our thinking toward the role of caspases in sepsis in terms of inflammation, cellular apoptosis and organ injuries.

## Caspase Functions

### Apoptosis

**Figure 1:**
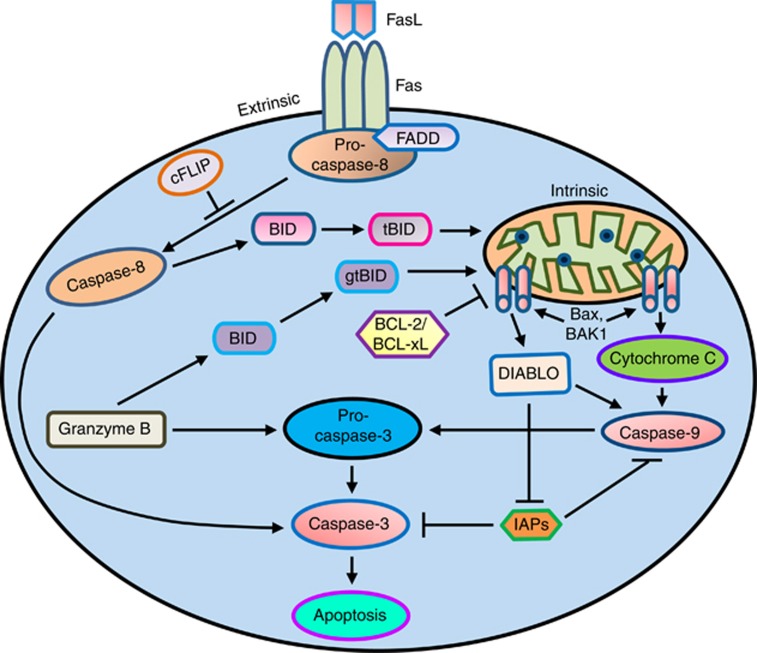
Apoptosis initiation by caspases: apoptosis can be initiated by the two major pathways involving initiator and effector caspases. The death-receptor (extrinsic) pathway acts through caspase-8 while the mitochondrial (intrinsic) pathway involves caspase-9. Both pathways converge to activate the effector caspase-3, which act on the death substrates. Caspase-8 can cleave the BH3-only protein Bcl-2 interacting domain death agonist (BID), forming a truncated BID (tBID), which can activate the intrinsic apoptotic pathway. Granzyme B is a cytotoxic cell proteinase-1, which directly cleaves and activates pro-caspase-3. Granzyme B can also cleave BID, resulting in granzyme tBID, which can activate the intrinsic apoptotic pathway. In addition to the caspases, cell death is regulated by Bcl-2 and inhibitor of apoptosis (IAP) protein families. Bcl-2, Bcl-X_L_ proteins are thought to regulate the mitochondrial permeability transition, thereby inhibiting cytochrome *c* release, while BAX and Bcl-2-antagonist/killer 1 (BAK-1) promote cytochrome *c* release, causing caspase-9 activation, which then leads to the activation of caspase-3 and promotes apoptosis. The IAP proteins act downstream to prevent processing of initiator caspase-9, and inhibiting the activity of the effector caspase-3. Pro-apoptotic mitochondrial factor, DIABLO (direct IAP protein-binding protein with low pI; also known as SMAC) is released and contributes to apoptosis by activating caspase-9 or inhibiting IAPs. cFLIP (cellular FLIP/caspase-8 inhibitor protein) acts as a negative regulator of the activation of caspase-8

Caspases are essential for apoptosis and drive two distinct pathways (Figure 1). The extrinsic apoptosis pathway is activated through the binding of a ligand to a death receptor (e.g. FAS), which in turn leads with the help of the adapter proteins (FAS-associated death domain (FADD)/TRADD) to recruitment, dimerization and activation of caspase-8. Active caspase-8 then either initiates apoptosis directly by cleaving and thereby activating executioner caspases (−3, −6 and −7), or activates the intrinsic apoptotic pathway through cleavage of BID to induce efficient cell death.^10^ The intrinsic or mitochondrial apoptosis pathway can be activated through various cellular stresses that lead to cytochrome *c* release from the mitochondria and the formation of the apoptosome comprised of APAF1, cytochrome *c*, ATP and caspase-9, resulting in the activation of caspase-9. Active caspase-9 then initiates apoptosis by cleaving and thereby activating executioner caspases.^10, 11^

### Pyroptosis

**Figure 2:**
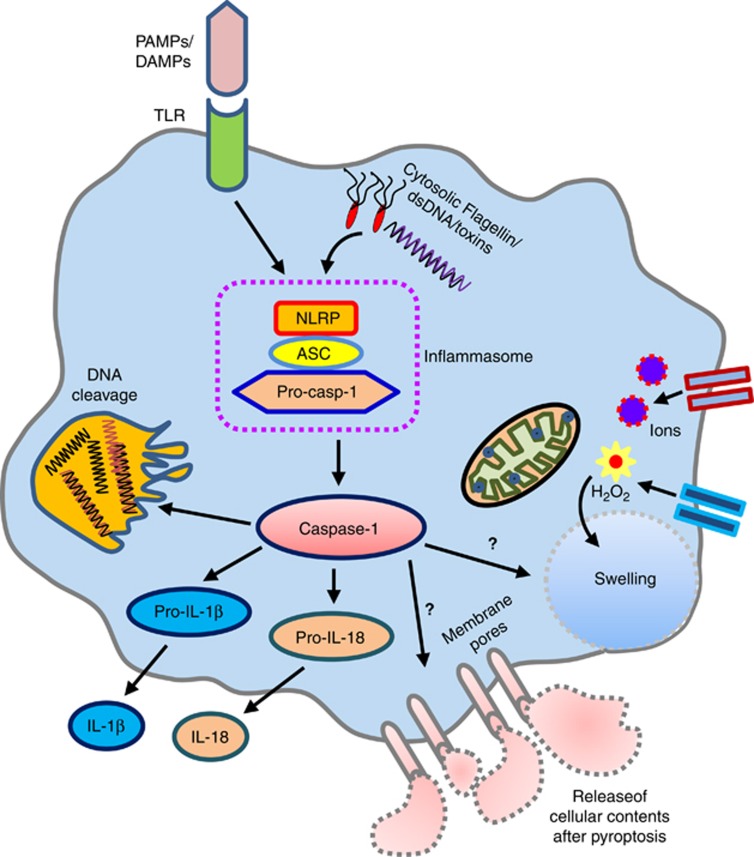
Caspase-1-induced pyroptosis: activation of the caspase-1 by inflammasome through toll - like receptors (TLRs) mediated by pathogen-associated molecular patterns (PAMPs) and damage-associated molecular patterns (DAMPs) or with the intracellular/extracellular substances, for example, cytosolic flagellin/dsDNA/toxins, trigger pyroptosis, a programmed lytic cell-death phenomenon. However, how caspase-1 directly promotes the formation of membrane pores and cell swelling is not well-understood

Pyroptosis is a form of programmed lytic cell death associated with antimicrobial responses during inflammation.^12^ Pyroptotic cell death elicits inflammation due to release of cytosolic contents such as ATP, high mobility group box-1 (HMGB-1) and IL-1*α*, and is subsequently accompanied by processing of inflammatory cytokines such as IL-1*β* and IL-18.^13, 14^ In contrast to apoptosis, pyroptosis is established as inflammasome-dependent cell death, executed following activation of caspase-1 or mouse caspase-11 (Figure 2).

### Necroptosis

**Figure 3:**
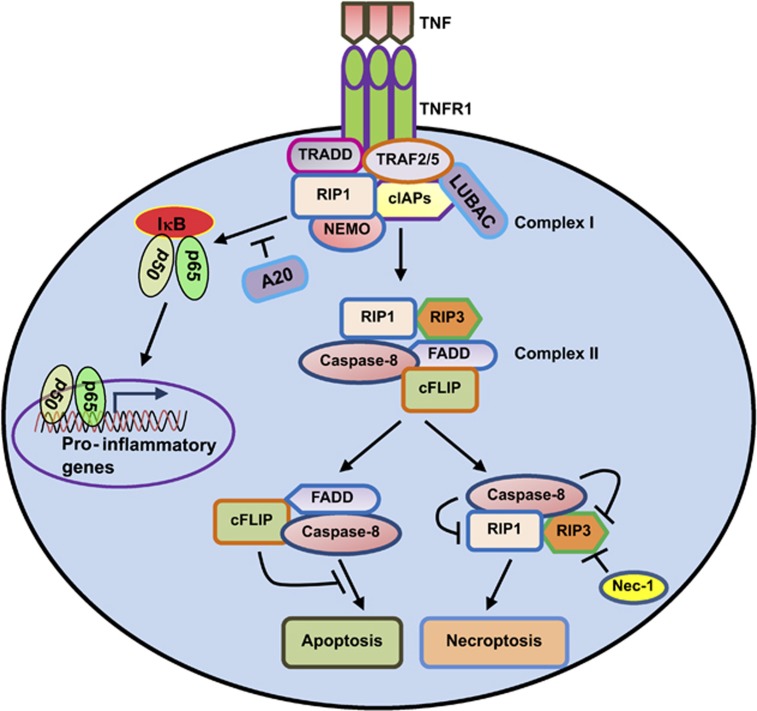
RIP1/3-mediated necroptosis: tumor necrosis factor (TNF) binding to its trimeric receptor, TNF receptor-1 (TNFR1) leads to a conformational change to generate TNFR complex I, which includes TNF receptor-associated death domain (TRADD), receptor-interacting protein 1 (RIP1; also known as RIPK1), cellular inhibitor of apoptosis proteins (cIAPs), TNF receptor-associated factor 2 (TRAF2) and TRAF5. Upon TNFR1 activation, linear ubiquitin chain assembly complex (LUBAC) promotes the recruitment and ubiquitination of the IKK-complex component, nuclear factor-*κ*B (NF-*κ*B) essential modulator (NEMO, also known as IKK*γ*). Ubiquitination of NEMO by LUBAC leads to NF-*κ*B phosphorylation, activation and translocation into the nucleus for inducing pro-inflammatory gene expression. A20 acts as a negative regulator of NF-*κ*B activation. In parallel, LUBAC also assures the linear polyubiquitinylation of RIP1, therefore preventing the exposition of the RIP1-assembly region that is required for complex II formation. Similarly, cIAPs is also involved in the polyubiquitinylation of RIP1. If RIP1 is deubiquitinylated by LUBAC inactivation, RIP1 will bind to RIP3 and activate the downstream cascade of necroptosis. Normally, caspase-8 triggers apoptosis by activating the classical caspase cascade. It also cleaves, and hence inactivates, RIP1 and RIP3. If caspase-8 is blocked by pharmacological or genetic interventions, RIP1 and RIP3 become phosphorylated by an unidentified kinase and engage the effector mechanisms of necroptosis. Necrostatin-1 (Nec-1) has been named for its ability to block necroptosis. Nec-1 is an allosteric inhibitor of RIP1 kinase activity

Different cellular stimuli (e.g., TNF, FAS ligand, TRAIL ligand, double-stranded RNA, interferon-*γ* (IFN-*γ*), ATP and pathogens) have been shown to induce necrosis that follows defined signaling events reminiscent of a cell-death program^15^ (Figure 3). Necroptosis can be defined as cell death mediated through a pathway that depends on the receptor-interacting protein kinase 1 (RIPK1 or RIP1)–RIPK3 complex and that can be inhibited by Necrostatin-1 (Nec-1).^16^ RIPK3 or RIP3 can also form complexes with DNA-dependent activator of IFN regulatory factor and the adaptor molecule TIR domain-containing adaptor-inducing IFN-*β*, leading to RIPK3-dependent programmed necrosis.^16, 17^

Mixed lineage kinase domain-like protein (MLKL) has recently been identified to interact with RIPK3 and become phosphorylated during TNF-induced necroptosis.^18, 19^ MLKL is indispensable for immune cell development. Embryonic fibroblasts and macrophages of MLKL-deficient mouse showed resistance to necrotic but not apoptotic stimuli.^19^ During TNF-induced necroptosis MLKL forms a trimer through its amino-terminal coiled-coil domain and locates to the plasma membrane. The trimerization requires both RIPK1 and RIPK3 because treating the cells with Nec-1, a RIPK1 inhibitor, or knocking down RIPK3 prevented the trimerization of MLKL.^19^ The membrane localization of MLKL is essential for Ca^2+^ influx, which is an early event of TNF-induced necroptosis. Further works on MLKL also reveals that the transient receptor potential melastatin related 7 (TRPM7) is a MLKL downstream target for the mediation of Ca^2+^ influx and TNF-induced necroptosis.^19^

### Inflammation

**Figure 4:**
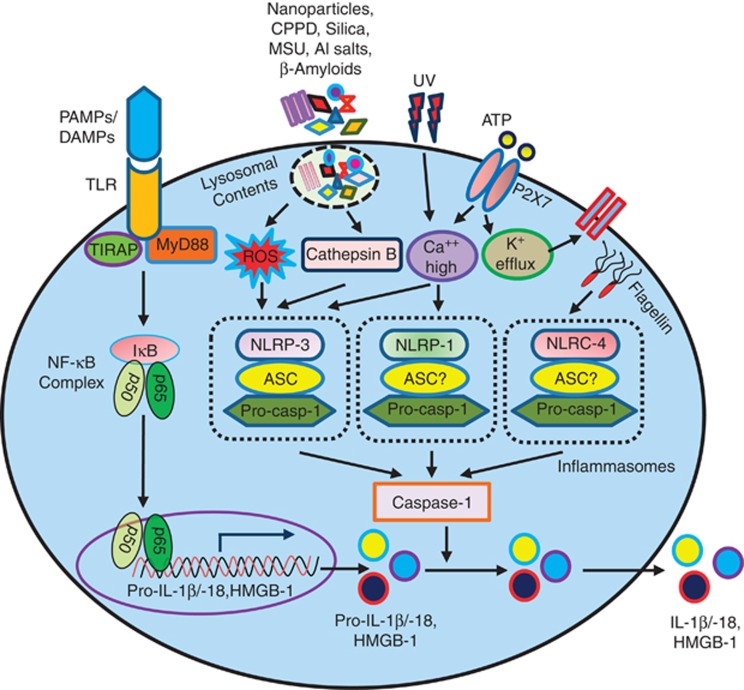
Caspase-1 activation by inflammasomes leads to pro-inflammatory cytokine production: Inflammasomes can be triggered by various stimuli. Pathogen-associated molecular patterns (PAMPs) or damage-associated molecular patterns (DAMPs) through their pattern recognition receptors (PRRs) activate NLRP3 inflammasome and induce IL-1*β* and IL-18 secretion in the presence of ATP. In addition, external ATP, which is considered as a danger signal, causes the opening of the P_2_X_7_ receptor, leading to the release of intracellular potassium and accumulation of increased amount of Ca^++^. Both pattern recognition receptors (PRRs)/TLRs- and P_2_X_7-_mediated pathways work jointly to activate inflammasome complex. Besides PAMPs, the NLRP3 inflammasome can also be activated by molecules contained in the lysosomes, including crystalline and particulate substances, with the concurrent signaling driven by ATP-P_2_X_7_ axis. In presence of ATP, the crystals of uric acid and reactive oxygen species (ROS) are known to activate NLRP3 inflammasome formation, which leads to the recruitment and activation of caspase-1. IL-1*β* and IL-18 secretion is regulated in a two-step manner. Their transcription is induced by Toll-like receptors, which detect extracellular microbe-associated molecular patterns. After transcription, pro-IL-1*β* and pro-IL-18 are held in reserve in the cytosol unlike other cytokines and chemokines, which are secreted after production. Inflammasomes regulate a proteolytic processing step that is required for IL-1*β* and IL-18 to be secreted. Mature form of high mobility group box-1 (HMGB-1) is also processed and secreted through the inflammasome-mediated pathway. In addition, there are several other commonly known NLRP inflammasomes, such as NLRP1 and NLRC4, which can activate caspase-1. It has been shown that K^+^ efflux appears to be essential for NLRP1 activation. On the other hand, the NLRC4 inflammasome becomes activated by cytosolic flagellin. The adaptor protein apoptosis-associated speck-like protein containing a caspase-recruitment domain (CARD) (ASC) is required in inflammasome complexes to bridge the interaction between upstream PRRs and inflammatory caspases through its amino-terminal pyrin domain (PYD) and carboxy-terminal CARD, respectively. However, the involvement of ASC in NLRP1 and NLRC 4 inflammasome formation is less easily understood

In humans caspase-1, -4, -5 and -12 and in mouse caspase-1, -11 and -12, as well as their pro-apoptotic counterparts, are produced as inactive forms in resting cells.^11, 20^ After cellular stimulation via engagement of pattern recognition receptors (toll-like receptors (TLRs)) with their respective pathogen-associated molecular patterns and damage-associated molecular pattern and also by the intracellular crystals, silica and lysosomal contents, they are activated through the formation of a cytosolic complex called inflammasome^20^ that ultimately engage caspases to help processing and releasing IL-1*β* and IL-18 (Figure 4).

### Proliferation

Functional analysis of conditional caspase-deficient mice demonstrates caspase-dependent extensive cellular responses such as cell differentiation, proliferation and nuclear factor-*κ*B activation.^21, 22^ Theoretically the role of caspase-8 in cellular proliferation is hard to explain, because of the fact that caspase-8 activation can either promote apoptotic or non-apoptotic signal depending on its extent of activation/inactivation. However, growing body of evidence demonstrates an essential role for caspase-8 in the proliferation of immune cells.^23, 24, 25, 26^ It executes cellular apoptosis by activating executioner caspases (-3, -6 and -7), as well as through the intrinsic pathway by cleaving the BID protein. The adaptor protein, FADD recruits and activates caspase-8 to initiate apoptosis and this pathway can be blocked by FLICE-like inhibitory protein long (FLIP_L_).^10^ Despite their role in cell death, FADD, caspase-8 and FLIP_L_ are all essential for embryonic development, suggesting that they are also having a pro-survival role. It has recently been shown that during development, FADD and caspase-8 promote survival by suppressing the function of RIPK1- and RIPK3-mediated necroptosis.^27, 28^ Patients bearing inactivating mutations in caspase-8 had impaired proliferation of T, B and natural killer cells.^25^ Caspase-8 has an important role in maintaining the fine tuned balance between cellular apoptosis and proliferation. Although the blocking of caspase-8 function may abrogate cellular apoptosis, it can also exaggerate necroptosis mediated by uncontrolled RIPK1 and RIPK3 activation. In a review article, Lamkanfi *et al.*^22^ nicely explained how c-FLIP_L_ levels modulate caspase-8 activation and downstream signaling. Beside this, caspase-8 also participates in differentiation of monocytes into macrophages and placental villous trophoblasts.^29, 30^ In contrast to caspase-8 functions for cell survival, caspase-3 on the other hand may have the opposite effect, as B cells lacking caspase-3 showed increased rate of proliferation after mitogenic stimulation.^31^

## Apoptotic Caspases in Sepsis

Studies indicate that both the extrinsic and intrinsic pathways are likely to be involved in sepsis-induced lymphocyte apoptosis.^32^ During sepsis, caspase-8 can be activated by ligands of the different death receptors, like FAS ligand^32, 33, 34^ or TNF-*α*.^6^ Similarly, in another sepsis study the mitochondrial pathway was shown to be activated by BID, a pro-apoptotic member of the B-cell lymphoma-2 (Bcl-2) family protein.^35^ Immunohistochemical studies of the spleen of patients who died of sepsis showed findings that are consistent with activation of this pathway.^36, 37^

In addition to the processing of IL-1*β*, caspase-1 may execute apoptosis during sepsis. A recent study has identified significantly higher amount of circulatory microvesicles (MVs) released from the monocytes of sepsis patients, which contained ample amount of caspase-1 and was capable of inducing apoptosis in healthy donor lymphocytes.^38^ In line with the above finding, depletion of MVs greatly diminished the apoptotic signals and restored the protective immune response against infection.^38^ Similarly, in a murine endotoxemia model, the role of caspase-7 has been shown clearly, where the caspase-7 knockout mice were resistant to LPS-induced lymphocyte apoptosis and were markedly protected from lethality independently of the excessive production of serum cytokines.^39^

## Inflammatory Caspases in Sepsis

Studies with inflammatory caspases in sepsis generated several intriguing views along with notable controversies. Sarkar *et al.*^40^ was the pioneer demonstrating a protective role by the caspase-1^−/−^ animals but not the IL-1^−/−^ and IL-1*β*/IL-18 double knockout mice against sepsis. Hence, the caspase-1 function in sepsis was independent of processing and releasing IL-1*β* and IL-18, in spite of having reduced levels of serum IL-1*β*, IL-18 and IL-6 in caspase-1^−/−^ mice during sepsis. Their study revealed a critical role of caspase-1^−/−^ mice in sepsis by protecting splenic B-lymphocytes from apoptosis, which might in turn restore natural antibody production by the B cells, preventing host from bacterial predisposition.^40^ However, Vanden Berghe *et al.*^41^ recently opposed that finding and made a strong statement by showing optimal protective outcomes against sepsis in terms of reducing systemic damage, cytokine levels and mortality in those mice, which had combined knocked-down of IL-1*β* and IL-18 genes, instead of those lacking only caspase-1. The study also revealed that caspase-7, a direct substrate of caspase-1, did not have any deleterious role in sepsis, in contrast to what was suggested previously.^39^ Even indeed, not only were the caspase-7^−/−^ mice sensitive to LPS doses, IL-1*β*/IL-18/caspase-7 triple knockout mice were not additively protected in comparison with IL-1*β*/IL-18 double knockout mice.^41^ The controversies that have been aroused might be due to the differences in mice genetic background, experimental setup, source and the doses of LPS to induce sepsis among individual labs. Moreover, the possibility of having caspase-11 inactivating passenger mutations, which we have discussed later at this section, could be another fact of this disputed area.^42^

Interestingly, it has been shown that reduced serum HMGB-1 levels in caspase-1-deficient mice correlated with their resistance to endotoxin, and HMGB-1 release required the inflammasome assembly and caspase-1 activation.^43^

In contrast to caspase-1, the hypothetical caspase-11-activating platform has been termed as the noncanonical inflammasome based on the finding that caspase-11 gene-targeted mice are critical for caspase-1 activation and IL-1*β* production in macrophages infected with gram-negative species.^44^ Later in a recent study it has been proved that macrophages stimulated with LPS can activate caspase-11 independent of the LPS receptor TLR-4.^45^ They have also reported that TLR-4^–/*–*^ mice primed with TLR-3 agonist polyinosinic : polycytidylic acid to induce pro-caspase-11 expression were as susceptible as the wild-type mice during endotoxemia.^45^ Similarly, Hagar *et al.*^46^ has recently showed that during endotoxemia, excessive caspase-11 activation can cause septic shock. They suggest that the contamination of cytoplasm by LPS is the signal that triggers caspase-11 activation in mice. Priming the caspase-11 pathway *in vivo* resulted in extreme sensitivity to subsequent LPS challenge in both wild-type and TLR-4-deficient mice, whereas caspase-11-deficient mice were relatively resistant.^46^ However, a recent report utilizing caspase-1 and caspase-3 null mice revealed the presence of an inactivating caspase-11 passenger mutation in the caspase-11 gene locus that is originated from the 129-derived embryonic stem (ES) cell line and is partially responsible for the resistance of caspase-1-null mice to endotoxin-induced shock.^42^ These results therefore indicate that the use of transgenic or knockout mice from different sources and undefined back crossing status may cause conflicting data in the field of sepsis, in this particular case due to the existence of mutant caspase-11 alleles originating from the 129 genetic background of the ES cell line. The use of conditional transgenic mice and their proper controls or transgenic mice generated with ES cells may resolve this issue.

In addition to the role of caspases in the processing of pro-inflammatory cytokine precursors to generate mature pro-inflammatory cytokines, there is now evidence of a role for caspase-12 in opposing or counter-balancing the pro-inflammatory response.^47^ Although the caspase-12-mediated endoplasmic reticulum-specific apoptosis and cytotoxicity has been reported earlier,^48^ a recent study reveals an essential role for caspase-12 in regulating inflammation in humans, where the Afro-American sepsis patients had a higher mortality rate compared with the other patients.^47^ The polymorphisms in caspase-12 gene, which results in the synthesis of either a truncated protein or a full-length caspase proenzyme caspase-12 long, were predominant in Afro-American descents, causing them to be more susceptible to endotoxemia due to inadequate immune response for cytokine production without effecting cellular apoptosis.^47^ Subsequent work in caspase-12-deficient mice has supported the above findings in humans, proving the fact that the mice that were deficient in caspase-12 had marked differences in cytokine production and improved survival in sepsis.^49^

## Pyroptotic Caspase in Sepsis

The role of caspase-1 in pyroptotic cell death in sepsis has been established from a study, which identifies a number of proteins that act as the substrates for caspase-1, for example, glycolysis pathway proteins.^50^ Sepsis activates caspase-1, which results in pronounced processing of the glycolysis pathway proteins in the macrophages, leading to cellular pyroptosis.^50^ In a recent study, Hu *et al.*^51^ indicated that the stimulation of macrophages with LPS and ATP induced the features of pyroptosis, including the expression of IL-1*β* mRNA and protein, activation of caspase-1, inflammasome formation and cell death.

Although it is likely that the blocking of cellular pyroptosis by inhibiting caspase-1 could be a promising therapeutic tool in sepsis, however, the recent work by Vandenabeele and co-workers^41^ showed that the caspase-1- and its amplifier-, caspase-11-deficient mice did not confer protection against a lethal TNF or cecal ligation and puncture (CLP)-induced sepsis, unless simultaneous targeting of IL-1*β* and IL-18 to get rid of sepsis severity. In line with the above findings, the latest article also precludes the need of caspase-1/3/7 for *Escherichia coli*-induced cellular pyroptosis, instead reliant on phosphatidylserine exposure prior to membrane rupture, therefore inhibition of caspase-1/11 may not exert complete protection against sepsis.^52^

A recent study identifies the novel role of caspase-11 to trigger pyroptosis in sepsis.^12^ Caspase-11 discriminates cytosolic from vacuolar/extracellular bacteria by a distinct mechanism that detects bacteria escaping from the phagosome into the cytosolic compartment, and is therefore a critical defense system against cytosolic bacteria. Aachoui *et al.*^12^ revealed that the caspase-11 knockout mice were sensitive to infection by cytosolic bacteria, but resistant to LPS sepsis. Further studies to explore the mechanism by which cytosolic bacteria are detected will provide additional insights underlying septic shock.

## Rip Kinase–Caspase-Dependent Necroptosis in Sepsis

In a recent study, Duprez *et al.*^53^ revealed that the RIPK-dependent increased necroptosis perturbed lethal systemic inflammatory response syndrome. According to their study, deletion of RIPK3 conferred protection against lethal SIRS and reduced the amounts of circulating damage-associated molecular patterns. Similarly, pretreatment with the RIPK1 inhibitor, Nec-1, provided a similar effect. These results suggest that RIPK1–RIPK3-mediated cellular damage by necrosis drives mortality during TNF-induced SIRS. Moreover, using a clinically relevant model, they have also suggested that RIPK3 deficiency also protected against CLP-induced sepsis, underscoring the clinical relevance of RIPK inhibition in sepsis. Towards linking to the caspases involvement into this RIPK-dependent cellular necrosis in sepsis, they have noticed that RIPK1- and RIPK3-dependent necroptosis in cells in sepsis could be inhibited by caspase-8, but not by the executioner caspases, caspase-3 and -7.^53^ Nevertheless, Wu *et al.*^18^ found discrepancy with the above report mentioning that neither MLKL nor RIPK3 deficiency could protect against polymicrobial sepsis in mice. The discrepancies of these two reports establishing the role of RIPK3 in sepsis might be due to various natures of luminal bacteria that were unique to the experimental animals housed in a particular animal facility. Therefore, further study to investigate whether or not the distinct resident cecal bacterial groups might elicit differential host responses in septic shock would resolve this debate. MLKL and RIPK3 deficiency can abrogate necroptosis without affecting other cell death and necrosis machineries of cells. Therefore, the possible explanation of extensive tissue damage and mortality that happened in MLKL or RIPK3 mice could be due to exaggerated nature of other types of cell-death pathways.^18^

For authentic interpretation of the Nec-1-based findings in experimental disease models, one should consider several critical issues on its specificity and activity. Apart from RIPK1, Nec-1 was also shown to inhibit indoleamine-2,3-dioxygenase.^54^ Takahashi *et al.*^55^ recently carried-out a comparative study using three Nec-1 analogs: (i) Nec-1, the active inhibitor of RIPK1, (ii) Nec-1 inactive (Nec-1i), its inactive variant and (iii) Nec-1 stable form (Nec-1s). Because of the short half-life of the Nec-1, a much improved analog, Nec-1s, was generated by chemical optimization of Nec-1.^56^

## Therapeutic Potentials of Caspase Inhibitors and Others

### Caspase inhibitors

Braun *et al.*^57^ were the first to report that pan caspase inhibitor N-benzlyoxycarbonyl-valylalanyl-aspartyl-fluoromethylketone (zVAD.fmk) that provided significant neuroprotection by decreasing hippocampal neuronal death in a rabbit model of pneumococcal meningitis. Shortly after, Hotchkiss *et al.*^58^ showed an improved survival in sepsis using zVAD.fmk by decreasing lymphocyte apoptosis in mice. They have also revealed that apart from the polycaspase inhibitor, zVAD.fmk, the selective caspase-3 inhibitor L-826,791 (M-791) has beneficial effects in sepsis through the inhibition of lymphocyte apoptosis.^9^ Similarly, in a recent study of a CLP model of sepsis using the VX-166, a broad spectrum caspase inhibitor showed beneficial effects for the treatment of sepsis.^59^ VX-166 showed potent anti-apoptotic and anti-inflammatory effects by inhibiting the release of IL-1*β* and IL-18 through attenuating the caspase-1 pathway in sepsis. Studies by Wesche-Soldato *et al.*^60^ showed that the siRNA directed against the caspase-8 gene decreased apoptosis and improved survival in the CLP model of sepsis. Similarly in a recent study, gene silencing of caspase-8 and caspase-3 with siRNAs provided profound protection against polymicrobial endotoxic shock through the prevention of vascular endothelial cell apoptosis.^61^ Although controversies exist, caspase-1^−/−^ animals were shown to have better protection against sepsis through the inhibition of splenic lymphocyte apoptosis, independent of attenuating IL-1*β* and IL-18 production.^40^ On the other hand, it is worth mentioning that the combined targeting of the caspase-1 downstream targets, IL-1*β* and IL-18 by using their knockout mice or neutralizing strategies using the IL-1*β* receptor antagonist, anakinra and anti-IL-18 antibodies, conferred complete protection against endotoxin-induced lethality.^41^ In line with the findings by Vanden Berghe *et al.*^41^ , recently Giamarellos-Bourboulis *et al.*^62^ found that both pro-caspase-1 and activated caspase-1 were markedly decreased in patients with sepsis, and blocking caspase-1 inhibited the release of IL-1*β* in healthy volunteers, an effect that was lost in septic patients. In addition, they have also noticed that the ligand stimulation significantly induced NLPR3 inflammasome activation, as well as IL-1*β* production in healthy controls but not in septic patients, suggesting that the downregulation of caspase-1 and defective IL-1*β* production are important immunological features in sepsis. Another study with caspase-1-deficient mice showed higher mortality to *Salmonella typhimurium* infection compared with the wild-type animals, indicating caspase-1 to be an essential molecule for host defense against bacterial infection that concludes with the fact that the caspase-1 substrates are required at distinct times and anatomical sites.^63^ Similarly, the beneficial survival outcome can be attained in the caspase-3^−/−^, caspase-11^−/−^ and caspase-1^−/−^/11^−/−^ mice strains with respect to protection from cellular apoptosis.^9, 42, 44^ Recent report showed that the apoptotic executioner caspase-7 was activated in the splenocytes of LPS-injected mice, suggesting a role for caspase-7 in lymphocyte apoptosis.^39^ Therefore, caspase-7-deficient mice were resistant to LPS-induced lymphocyte apoptosis and were markedly protected from LPS-induced lethality independently of the excessive production of serum cytokines. These results reveal for the first time a nonredundant role for caspase-7 *in vivo* and identify caspase-7 inhibition as a component of the mechanism by which caspase inhibitors protect from endotoxin-induced mortality.^39^ However, Duprez *et al.*^53^ recently examined the role of apoptotic executioner caspases (caspase-3 and -7) and the inflammatory caspase-1 in a cellular model, as well as in an *in vivo* mouse model of TNF-induced toxicity, in which the authors showed that the depletion of neither the executioner caspases nor caspase-1 had any effect in the cellular model or in TNF-induced SIRS, ruling out the involvement of caspase-dependent apoptosis and caspase-1-mediated inflammation and/or pyroptosis in sepsis.^53^ The outcomes of caspase-, MLKL-, as well as RIPK-knockout mice in sepsis are shown in Table 2.

In addition to caspase-1, neutrophil serine proteases such as proteinase 3 (PR3), elastase or cathepsin-G, can process IL-1*β* in a caspase-independent pathway.^64, 65^ As both caspase-1 and PR3 are considered to be potential targets in inflammation, determining the role of PR3 in sepsis is crucial for the development of novel anti-IL-1*β* therapies. In this regard, mice lacking both caspase-1 and PR3 were protected from inflammation, suggesting that during acute phase where neutrophil infiltrate predominates PR3 serves as the source for activated IL-1*β*.^66^ Thus, the dual inhibition of caspase-1 and serine proteinases will be the method of choice for an effective therapy in ameliorating inflammation. However, the role of serine proteinases in sepsis, is yet to be determined.

### Intracellular/death receptor blocking strategies

Studies showed that lymphocytes from the Bcl-2-transgenic mice were found to be resistant to sepsis-induced apoptosis, and that these mice had a significant improvement in survival compared with the wild-type controls.^67, 68^ Similarly, the strategies employed to overexpress the survival factor Akt decreased sepsis-induced lymphocyte apoptosis in a Bcl-2-independent manner and improved survival.^69^ Recent studies have also shown that inhibiting the FAS-mediated apoptotic pathway by using FASL-deficient mice, administering a FAS fusion protein to inhibit FAS-induced signaling or by siRNA knockdown of FAS reduced mortality in murine polymicrobial sepsis.^60, 70, 71, 72^ Indeed, injection of siRNA directed against FAS after the induction of CLP improved survival by 50%.^60^

In addition to caspases, other proteases might also be involved in mediating apoptosis.^73, 74^ Weaver *et al.*^75^ tested commonly utilized HIV protease inhibitors in a mouse CLP model of sepsis, which surprisingly decreased lymphocyte apoptosis and improved survival, even if post treatment of CLP. Recent studies have shown that large macromolecular cargos, proteins and peptides can be delivered intracellularly if conjugated to permeation peptides that are derived from transcriptional transactivator (Tat)-basic domains.^76, 77^ Hotchkiss *et al.*^78^ have shown that a Tat-BH4 conjugate had a high potent effect on decreasing sepsis-induced lymphocyte apoptosis *in vivo* in the mouse CLP model. Studies to determine whether the Tat-BH4 conjugate not only prevents sepsis-induced apoptosis but improves survival are currently underway.

## Potential Pitfalls of Caspase Inhibitors Introduce Immunomodulatory Therapies in Sepsis

The results from using caspase inhibitors have not been consistent, possibly because of the variability of the CLP model and/or the difficulty in successfully inhibiting caspase activation. It has been determined that only a small amount of activated caspase-3 is sufficient to initiate apoptotic cell death.^79^ Therefore, it is necessary to have a high degree of inhibition of caspases to prevent cell death. This requirement presents great therapeutic challenges owing to the need for persistent and nearly complete caspase blockade. Ample number of literature supports the notion that caspases have several other functions apart from their role as cell-death proteases and regulators of inflammation, which include lymphocyte activation, proliferation and protective immunity.^4, 12, 29, 78, 80^ Indeed, patients with defects in caspase-8 are immunodeficient and suffer from recurring infections,^26^ and caspase-6 has an essential role in B-cell proliferation.^80^ Therefore, blocking caspases might have some beneficial effects in decreasing apoptosis in sepsis but these could be counterbalanced by their adverse effects on the ability of the patient to mount an effective immune response. There is a report indicating inhibition of caspases might even induce hyper-acute TNF-*α*-induced shock in certain situations.^81^

Cellular inhibitor of apoptosis protein (cIAP2) has been shown to be a key regulator of the innate immune response.^82^ Therefore, studies involving cIAP2-deficient mice were highly resistant to endotoxin-induced mortality, with decreased secretion of pro-inflammatory cytokines following endotoxin challenge.^82^ Ironically, the cIAP2^−/−^ mice not only display an attenuated inflammatory response but also become susceptible to Fas-, platelet-activating factor- and LPS-induced death.^82^ Therefore, manipulation of cIAP2 expression may not be a promising tool to combat against endotoxemia because of its role in cellular homeostasis. It has been reported that the ES cell lines derived from the 129 mouse strain carry an inactivating mutation within the caspase-11 locus, therefore, if the 129 ES cells are used to target genes closely linked to caspase-11, the resulting mice may also carry the caspase-11 deficiency as a passenger mutation.^44^ Paradoxically, Kenneth *et al.*^83^ showed that the mice originated by targeting c-IAP1 in a 129-derived ES cell line contained an added layer of complexity if used to investigate the roles of c-IAP1 in innate immunity and programmed cell death because of a concealed defect in the caspase-11 gene. Therefore, targeting cIAPs for controlling innate immune response may require additional concern to overcome the pseudo effects caused by caspase-11 passenger mutations.

As described in section 6, RIPK1- and RIPK-3-mediated necroptosis drives increased mortality during TNF-induced SIRS an effect that could be blocked by the presence of caspase-8, which promoted RIPK1 and/or RIPK3 cleavage and inhibited necroptosis.^53^ Considering this fact, using caspases pan inhibitors like zVAD.fmk and VX-166 or specific inhibition of caspase-8 may trigger necroptosis, which may further deteriorate sepsis. In line with this statement Duprez *et al.*^53^ showed that the mice pre-treated with zVAD.fmk did not show protection against TNF-induced SIRS, unless Nec-1 was administrated into them to get better survival outcome, which therefore corresponds to the previous finding by Cauwels *et al.*^81^ showing co-administration of zVAD-fmk sensitized mice to TNF-induced SIRS.

The efficacy of zVAD.fmk to attenuate cellular apoptosis in sepsis was found to be most effective only if it was given before inducing the sepsis in mice.^58^ Since most sepsis patients admitted to the ICU are already severely lymphopenic, the caspase inhibitors may not likely be useful in humans. At the late stage of sepsis, patients suffer from severe immunosuppression due to higher rate of lymphocyte apoptosis and tolerance.^6^ Ideal immunomodulatory therapy directed against sepsis can boost overall patient immunity, drive lymphocyte effector functions, decrease lymphoid apoptosis and ultimately mitigate the development of immune suppression, which has been often associated with onset of secondary infections and death among critically ill patients.^84^ Recent studies from animal sepsis models and patients suggest that cytokines such as IL-7, IL-15, granulocyte macrophage colony-stimulating factor, as well as co-inhibitory molecule blockades, like anti-programmed cell-death receptor-1 and anti-B and T lymphocyte attenuator, have beneficial outcome in alleviating the clinical morbidity associated with sustained sepsis.^84, 85, 86^

## Conclusion and Perspectives

**Figure 5:**
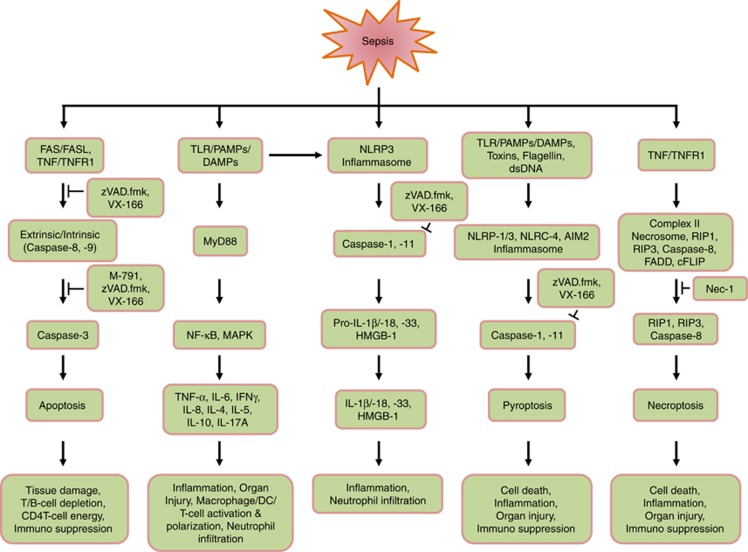
Role of caspases and their inhibitors in sepsis: schematic representation indicating the involvement of caspases in sepsis is shown. The pan caspase inhibitors, zVAD.fmk, and VX-166, and the caspase-3-specific inhibitor, M-791, show potential beneficial outcome in sepsis. Necrostatin-1 (Nec-1) serves as the inhibitor of RIPK1

The role of caspases and their inhibitors in sepsis to cause and protect from apoptosis, inflammation, pyroptosis and necroptosis has been summarized in Figure 5. Besides the conventional strategies utilizing the caspase inhibitors and gene knockout mice to block apoptosis, several other therapeutic regimens may have pivotal roles in protection from apoptosis involving the cellular machineries. Our recent studies utilizing recombinant milk fat globule-epidermal growth factor-factor 8 (rMFG-E8) provided striking beneficial findings in sepsis, cerebral ischemia and acute lung injury by inhibiting caspase-3-mediated cellular apoptosis and upregulating Bcl-2 expression.^87, 88, 89^ Further study awaits to determine which specific immune cell populations are protected from apoptosis during the treatment of rMFG-E8 in sepsis. Our existing article deals with the literature review demonstrating the current progress and prospects of caspase studies in sepsis caused by infectious process. Nonetheless, several septic incidents originated from non-infectious events as occurred after trauma, hemorrhage and ischemia are quite indistinguishable from infectious sepsis and generate similar prognosis. Future summarization of the role of caspases in non-infectious sepsis by periodical revisiting will additionally improve our understanding toward delineating sepsis pathobiology and novel therapeutics targeting caspases.

## Figures and Tables

**Table 1:** Categorized functions of caspases

**Cellular events**	**Caspases**	**References**
Apoptosis	Caspase-2, -3, -6, -7, -8, -9 and -10	^11^
Inflammation	Human: caspase-1, -4, -5 and -12; Mouse: caspase-1, -11 and -12	^12, 20^
Pyroptosis	Caspase-1 and -11	^12, 13, 14^
Necroptosis	Caspase-8 as negative regulator	^16, 53^
Proliferation	Caspase-6 and -8	^22, 23, 24, 25, 26^
Unspecified	Caspase-2, -10 and -14	^90^

**Table 2:** Effects of caspase-, MLKL- and RIPK3-deficient mice in sepsis

	**Outcomes in sepsis**	
**Knockout strains**	**Protective**	**Non-protective/deleterious**
Caspase-1	Improves survival; attenuates IL-1*β* production; decreases lymphocyte apoptosis.^40^	Higher mortality in *Salmonella typhimurium* infection.^63^ Not protective against TNF-*α*-induced SIRS.^53^
Caspase-3	Improves survival; less T- and B-cell apoptosis.^9^	No effect on survival in LPS-induced sepsis.^39, 42^ Does not improve survival against lethal dose of TNF-*α*.^53^
Caspase-7	Protects from LPS-induced lethality independently of the excessive production of serum cytokines; resistant to lymphocyte apoptosis.^39^	Not protective against lethal dose of LPS challenges.^41^ No survival benefit against TNF-*α*-induced SIRS.^53^
Caspase-11	Survival benefits; critical for caspase-1 activation; defects in IL-1*β* and IL-18 production.^44^	Partially protective against a lethal dose of TNF-*α*.^41^
Caspase-1/11 double KO	Improves survival; defects in IL-1*β* and IL-18 production.^44^	Partly improves survival against a lethal dose of TNF-*α*.^41^
Caspase-3/11 double KO	Significantly improves survival.^71^	
IL-1*β*/IL-18 double KO	Exerts potential survival benefits against LPS/TNF-*α*/CLP-induced sepsis.^41^	
IL-1*β*/IL-18/caspase-7 triple KO		No additional benefit in improving survival as compared with the IL-1*β*/IL-18 double KO.^41^
Caspase-12	Improves survival; Enhanced clearance of pathogens; Maintains IL-1*β* production.^49^	
MLKL	Blocks TNF-*α*-driven necroptosis.^91^	No effect on improving septic shock-induced animal death.^18^
RIPK3	Decreases cellular necroptosis; protects mice from TNF-induced SIRS; improves survival in CLP-induced sepsis.^53^	Does not protect from polymicrobial sepsis-induced mortality.^18^

Abbreviations: IL-1*β*, interleukin-1 beta; KO, knockout; MLKL, mixed lineage kinase domain-like protein; RIPK3, receptor-interacting serine–threonine kinase 3; SIRS, systemic inflammatory response syndrome; TNF-*α*, tumor necrosis factor alpha

## Abbreviations

CLP: cecal ligation and puncture
FADD: FAS-associated death domain
HMGB-1: high mobility group box-1
MFG-E8: milk fat globule-epidermal growth factor-factor 8
TLRs: toll-like receptors
zVAD.fmk: N-benzlyoxycarbonyl-valylalanyl-aspartyl-fluoromethylketone
